# Multiple Drugs Compete for Transport via the *Plasmodium falciparum* Chloroquine Resistance Transporter at Distinct but Interdependent Sites*

**DOI:** 10.1074/jbc.M114.614206

**Published:** 2014-11-06

**Authors:** Sebastiano Bellanca, Robert L. Summers, Max Meyrath, Anurag Dave, Megan N. Nash, Martin Dittmer, Cecilia P. Sanchez, Wilfred D. Stein, Rowena E. Martin, Michael Lanzer

**Affiliations:** From the ‡ Department of Infectious Diseases, Parasitology, Heidelberg University, Im Neuenheimer Feld 324, 69120 Heidelberg, Germany,; the § Research School of Biology, Australian National University, Canberra, Australian Capital Territory 0200, Australia, and; the ¶ Department of Biological Chemistry, Silberman Institute of Life Sciences, Hebrew University of Jerusalem, Jerusalem 91904, Israel

**Keywords:** Drug Resistance, Malaria, Membrane Transport, Parasitology, Transporter, Chloroquine, Mixed-type Inhibition, Quinine, Verapamil

## Abstract

Mutations in the “chloroquine resistance transporter” (PfCRT) are a major determinant of drug resistance in the malaria parasite *Plasmodium falciparum*. We have previously shown that mutant PfCRT transports the antimalarial drug chloroquine away from its target, whereas the wild-type form of PfCRT does not. However, little is understood about the transport of other drugs via PfCRT or the mechanism by which PfCRT recognizes different substrates. Here we show that mutant PfCRT also transports quinine, quinidine, and verapamil, indicating that the protein behaves as a multidrug resistance carrier. Detailed kinetic analyses revealed that chloroquine and quinine compete for transport via PfCRT in a manner that is consistent with mixed-type inhibition. Moreover, our analyses suggest that PfCRT accepts chloroquine and quinine at distinct but antagonistically interacting sites. We also found verapamil to be a partial mixed-type inhibitor of chloroquine transport via PfCRT, further supporting the idea that PfCRT possesses multiple substrate-binding sites. Our findings provide new mechanistic insights into the workings of PfCRT, which could be exploited to design potent inhibitors of this key mediator of drug resistance.

## Teaser

**Background:** Mutations in the chloroquine resistance transporter (PfCRT) change the susceptibility of *Plasmodium falciparum* to diverse antimalarial drugs.

**Results:** In addition to chloroquine, PfCRT transports quinine, quinidine, and verapamil, which bind to distinct but antagonistically interacting sites.

**Conclusion:** PfCRT is a multidrug carrier with a polyspecific drug-binding cavity.

**Significance:** These findings could be used to develop high affinity inhibitors of PfCRT.

## Introduction

Changes in the “chloroquine resistance transporter” (PfCRT)^5^ are associated with reductions in the susceptibility of the human malaria parasite *Plasmodium falciparum* to several important antimalarial drugs (1, 2). Initially identified as the main determinant of resistance to chloroquine (3), a synthetic 4-aminoquinoline and a mainstay in previous campaigns to eradicate malaria, mutations in PfCRT are now known to have wide-ranging effects on the parasite's sensitivity to an assortment of pharmacons (4). These include antimalarial drugs that share the quinoline scaffold (*e.g.* quinine, amodiaquine, and mefloquine) or that possess structural moieties present in quinoline drugs (*e.g.* lumefantrine and halofantrine) but also encompass a diverse range of compounds that have not been deployed as antimalarial treatments (4–12). Given that the activities of most of the antimalarials that currently serve as partner drugs in the artemisinin-based combination therapies are affected by mutations in PfCRT (1) and that all of the upcoming partner drugs are quinolines or quinoline-related, it is possible that PfCRT may evolve multidrug resistance capabilities that will render entire drug classes ineffective, including compounds that are in clinical and preclinical development. It is vital that we prolong the longevity and efficacy of the current quinoline drugs and also retard the emergence and spread of resistance to new antimalarials. A greater understanding of the mechanism by which PfCRT alters the parasite's susceptibility to diverse compounds could form the basis for antimalarial strategies that combat PfCRT-mediated drug resistance.

PfCRT is a member of the drug/metabolite transporter superfamily and displays the 2-fold pseudosymmetry typical of carriers (13). The transporter resides at the membrane of the parasite's digestive vacuole (3) and is thought to efflux drugs out of this organelle, away from their main target, the detoxification of heme arising from the digestion of host hemoglobin (1, 2, 14). Evidence of PfCRT functioning as a drug carrier has come from *in vitro* parasite assays as well as characterizations of PfCRT in heterologous expression systems. In the parasite studies, the efflux of radiolabeled drugs from parasite-infected red blood cells was linked to PfCRT (15–18), and PfCRT was also implicated in the drug-mediated efflux of protons from the digestive vacuole of chloroquine-resistant parasites (19–21). Moreover, heterologous expression of the Dd2 form of PfCRT (PfCRT^Dd2^) at endosomal membranes within *Dictyostelium discoideum* reduced the accumulation of chloroquine and quinine within these vesicles, consistent with the mutant protein mediating the transport of these two drugs (22, 23). Finally, a diverse range of chloroquine-resistant variants of PfCRT induced saturable chloroquine transport when expressed at the surface of *Xenopus laevis* oocytes (24, 25). By contrast, the wild-type form of the protein (found in chloroquine-sensitive parasites) did not exhibit chloroquine transport activity in this assay. A key advantage of the oocyte system is that it allows interactions with PfCRT to be studied directly and in isolation, without confounding effects such as the binding of drugs to heme or to other targets or transporters within the parasite-infected red blood cell.

Although it is now well established that chloroquine-resistant forms of PfCRT transport chloroquine, little is known about its ability to mediate the transport of other drugs or how the protein recognizes diverse compounds. For instance, it is unclear whether PfCRT accepts different drugs at a single site or at distinct sites. Several lines of evidence support the view that PfCRT possesses a single drug-binding site, with the lysine to threonine mutation at position 76 (K76T) playing a pivotal role in the binding and translocation of drugs (26, 27). Indeed, all chloroquine-resistant field isolates identified to date harbor a mutation at position 76, and reversal of the K76T mutation has been shown to abolish the transport of chloroquine via resistant forms of PfCRT (15, 24, 25) and to increase the parasite's susceptibility to a number of drugs, including chloroquine, quinine, and amodiaquine (28, 29). On the other hand, the fact that PfCRT variants of different geographic origins vary in both the number (typically 4–10 amino acid substitutions) and nature of the mutations they contain and that such variations may impart different drug responses (7, 8) suggests that a more complex interaction may exist between PfCRT and its drug substrates.

Here we investigated the interaction of PfCRT with chloroquine, quinine, quinidine, and verapamil. The latter compound can partially reverse chloroquine resistance *in vitro* (30). The PfCRT^Dd2^ variant of the protein (from the Southeast Asian strain Dd2, which is chloroquine-resistant and also exhibits reduced sensitivity to quinine) was expressed in *Xenopus* oocytes and shown to mediate the transport of radiolabeled chloroquine, quinine, quinidine, and verapamil. The results of an in depth kinetic examination of the inhibition of chloroquine or quinine transport by another drug suggest that PfCRT^Dd2^ possesses at least two distinct binding sites that antagonistically affect one another.

## EXPERIMENTAL PROCEDURES

### 

#### 

##### Ethical Statement

Ethical approval of the work performed with the *X. laevis* frogs was obtained from (i) the Australian National University Animal Experimentation Ethics Committee (Animal Ethics Protocol Number A2013/13) in accordance with the Australian Code of Practice for the Care and Use of Animals for Scientific Purposes and (ii) the Regierungspräsidium Karlsruhe (Aktenzeichen 35-9185 81/G-31/11) in accordance with the German “Tierschutzgesetz.”

##### Radiolabeled Drugs

[^3^H]Chloroquine (specific activity, 5–25 Ci mmol^−1^), [^3^H]quinine (specific activity, 10–20 Ci mmol^−1^), [^3^H]quinidine (specific activity, 5–25 Ci mmol^−1^), and [^3^H]verapamil (specific activity, 80 Ci mmol^−1^) were obtained from American Radiolabeled Chemicals or GE Healthcare.

##### Harvesting of Xenopus Oocytes and Expression of PfCRT

Adult female *X. laevis* frogs (purchased from NASCO) were anesthetized by submersion in a solution of 0.1% (w/v) ethyl 3-amino benzoate methanesulfonate and 1 mm NaCO_3_ for 15–20 min. Sections of the ovary were surgically removed and placed in Ca^2+^-free amphibian-adapted Ringer's solution (96 mm NaCl, 2 mm KCl, 1 mm MgCl_2_, and 5 mm HEPES, pH 7.5) supplemented with penicillin/streptomycin (10 mg/ml). The collagenous membrane that envelopes the oocyte lobes, as well as the individual oocytes, was removed by the addition of collagenase D (final concentration of 0.3 FALGPA units/ml). Following collagenase treatment, the oocytes were kept in Ca^2+^-replete amphibian-adapted Ringer's solution (96 mm NaCl, 2 mm KCl, 1 mm CaCl_2_, 1 mm MgCl_2_, and 5 mm HEPES, pH 7.5) supplemented with penicillin/streptomycin (10 mg/ml). Expression of the HB3 and Dd2 versions of PfCRT at the plasma membrane of *Xenopus* oocytes was achieved as described previously (24, 25). Briefly, cRNA was transcribed *in vitro* using a mMessage mMachine kit (Ambion) and microinjected into stage V-VI oocytes (30 ng/oocyte). Oocytes were incubated for 48–72 h at 18 °C in ND96 medium (96 mm NaCl, 2 mm KCl, 1.8 mm CaCl_2_, 1 mm MgCl_2_, 10 mm MES, and 10 mm Tris, pH 7.5), and water-injected oocytes served as a negative control.

##### Drug Transport Assays

Measurements of radiolabeled drug uptake were made over 1 h at 25 °C and in a ND96 medium that contained [^3^H]chloroquine (50 nm), [^3^H]quinine (62.5 nm), [^3^H]quinidine (62.5 nm), or [^3^H]verapamil (25 nm). Where specified, one or more unlabeled drugs were present at the indicated concentrations. The influx assays were terminated by removing the reaction medium and washing the oocytes three times with 3 ml of ice-cold ND96 buffer. Each oocyte was transferred to a separate scintillation vial and lysed by the addition of 5% sodium dodecyl sulfate (200 μl). The radioactivity in the sample solution was measured using a β-scintillation counter. The direction of radiolabeled drug transport in these assays is from the acidic extracellular medium (in most cases pH 6.0, and pH 4.0, 4.5, 5.0, or 5.5 where indicated) into the oocyte cytosol (pH ∼7.2), which corresponds to the efflux of drug from the acidic digestive vacuole (pH 5–5.5) into the parasite cytosol (pH 7.3). Water-injected oocytes and oocytes expressing PfCRT^HB3^ take up [^3^H]chloroquine to similar (low) levels via simple diffusion of the neutral species; this represents the “background” level of chloroquine accumulation in oocytes (see Ref. 24 for full data and a detailed discussion). PfCRT^Dd2^-mediated drug transport was calculated by subtracting the uptake measured in water-injected oocytes from that in oocytes expressing PfCRT^Dd2^. Where specified, statistical comparisons were made with Student**'**s *t* test for paired samples or with analysis of variance in conjunction with Tukey**'**s multiple comparisons test. The half-maximum inhibitory concentrations derived from Figs. 3, 5, and 9 (*A* and *B*) were obtained by least-squares fit of the equation, *Y* = *Y*_min_ + ((*Y*_max_ − *Y*_min_)/(1 + ([inhibitor]/IC_50_)*C*), where *Y* is PfCRT^Dd2^-mediated drug transport, *Y*_min_ and *Y*_max_ are the minimum and maximum values of *Y*, IC_50_ is the half-maximum inhibitory concentration, and *C* is a constant.

##### Discrimination between Models and Statistical Analyses of the Kinetic Data

Analyses of the kinetic data were performed using Sigma Plot version 12.5 and the software package “R” (31). The 16 different inhibition models (32) were globally fitted to the kinetic data using the least-squares method. The models were then ranked according to their Akaike information criterion difference (Δ*AIC_c_*) and their Akaike weight using a method described previously (33, 34). The two top-ranked models were compared using an F-test.

The Akaike information criterion (*AIC_c_*) of the *i*th model (*AIC*_*c*_^*i*^) was calculated according to Equation 1,

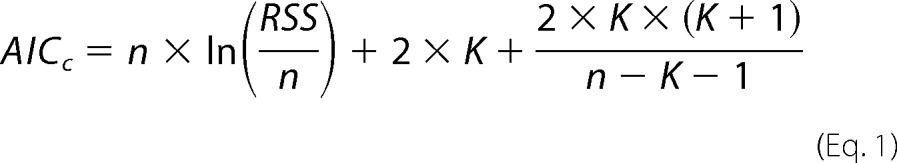
 where *RSS* is the residual sum of squares.

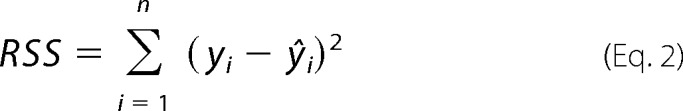
 Here, *n* is the total number of measurements used to perform the global fit, *y_i_* are the experimentally measured values, *ŷ_i_* are the values predicted by the model, and *K* is the number of parameters in the model. The ΔAIC*_c_* of the *i*th model (Δ*_i_*) was then calculated according to Equation 3,

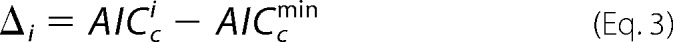
 where *AIC*_*c*_^min^ is the smallest *AIC_c_* value of all of the models tested. Models with Δ*AIC_c_* < 2 are equally plausible, models with 4 < Δ*AIC_c_* < 7 are considerably less likely, and models with Δ*AIC_c_* > 10 are considered unlikely (33).

For a more detailed evaluation of the plausibility of models, the Akaike weight (*w_i_*) was calculated according to Equation 4,

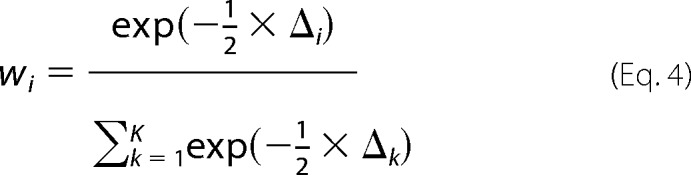
 where Δ*_i_* is as described above, Δ*_k_* is the *AIC_c_* difference of the *k*th model investigated, and *K* is the total number of models investigated. *w_i_* can be in the interval of 0 ≤ *w_i_* ≤ 1 and reports the plausibility of the model. The model with the highest Akaike weight (*i.e.* closest to 1) is considered to be the best fit to the data.

The *F* statistic was calculated according to Equation 5,

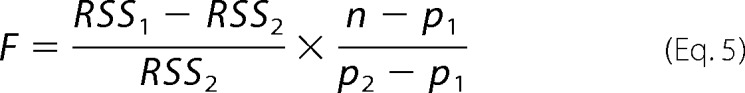
 where *RSS*_1_ and *RSS*_2_ are given by Equations 6 and 7,

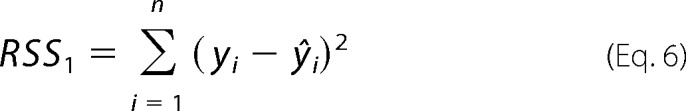


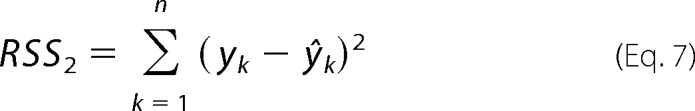
 where *RSS*_1_ is the residual sum of squares of the first model, *RSS*_2_ is the residual sum of squares of the second model, *y_i_* and *y_k_* are the experimentally determined values for the first and second models respectively, and *ŷ_i_* and *ŷ_k_* are the predicted values for the first and second models. *n* is the total number of data points, and *p*_1_ and *p*_2_ are the number of parameters present in the first and second models, respectively. The *p* value of the corresponding *F* statistics and its degrees of freedom were calculated using R. *p* < 0.05 indicates that the model with fewer parameters fits the data significantly better than the model with more parameters. All analyses of the kinetic data were performed using Sigma Plot version 12.5 or the software package R (31).

##### Equations Describing 16 Different Kinetic Models

The following kinetic equations were adapted from Segel (32). The concentration of substrate 1 is denoted as [S1] and the concentration of the second, inhibiting substrate is denoted as [S2]. *K*_S1_ and *K*_S2_ are the dissociation constants for the respective substrate-protein complexes. Full mixed-type (linear mixed-type) inhibition is given by Equation 8.

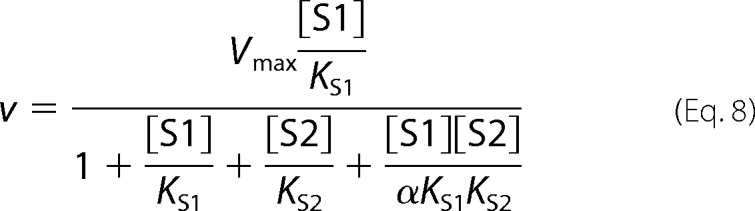
 Partial mixed-type (hyperbolic mixed-type) inhibition is given by Equation 9.

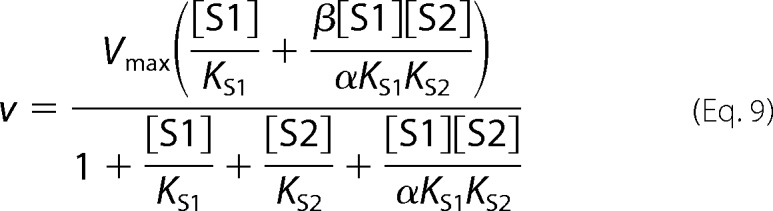
 Full competitive inhibition is given by Equation 10.

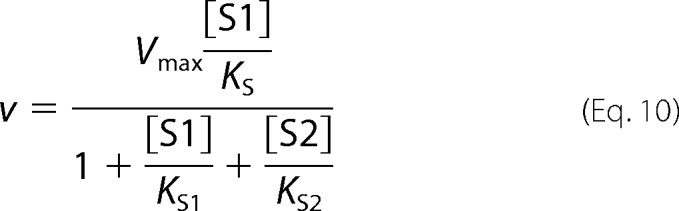
 Partial competitive inhibition is given by Equation 11.

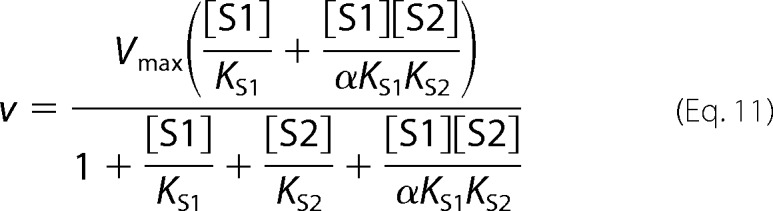
 Full noncompetitive inhibition is given by Equation 12.

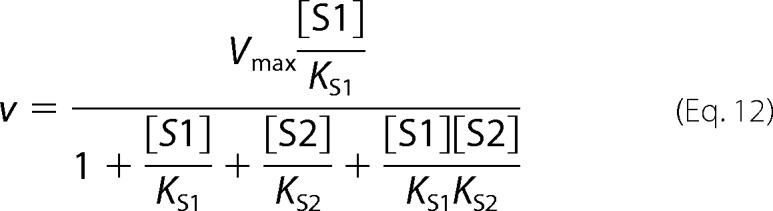
 Partial noncompetitive inhibition is given by Equation 13.

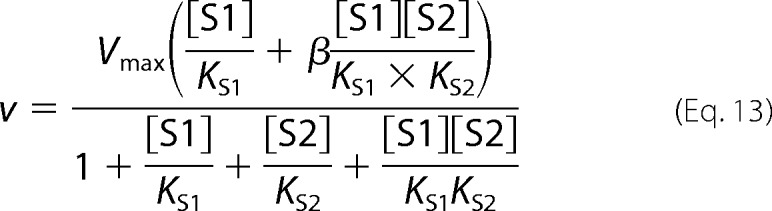
 Full uncompetitive inhibition is given by Equation 14.

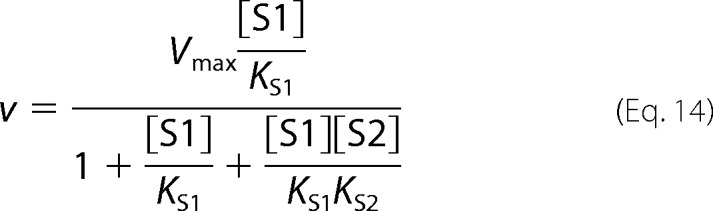
 Partial uncompetitive inhibition is given by Equation 15.

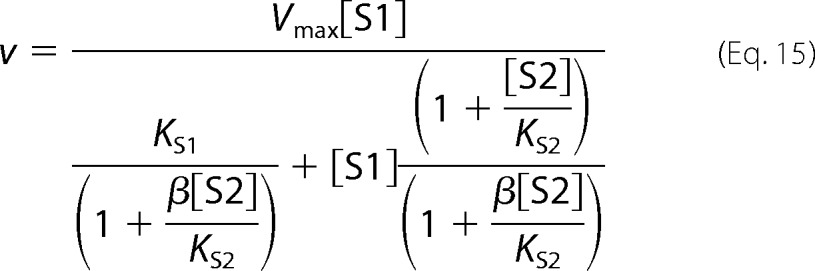
 Ligand exclusion is given by Equation 16.

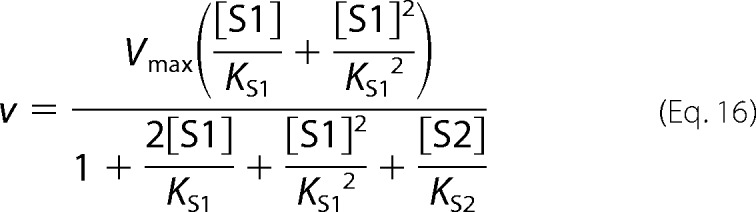
 Cooperative substrate binding is given by Equation 17.

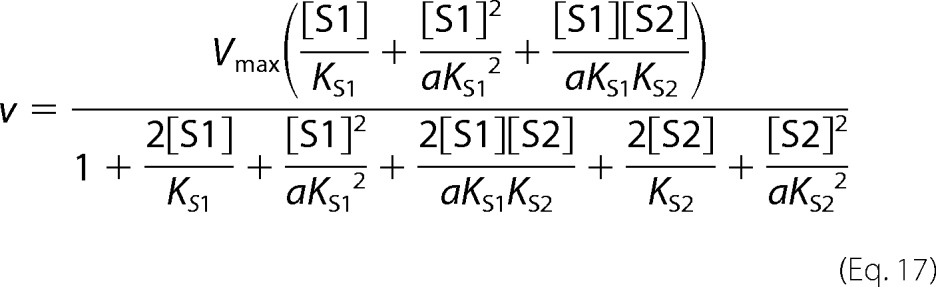
 In cooperative substrate binding, the inhibitor does not mimic the substrate. This case is asymmetric; hence, there are different equations for the substrate (Equation 18) and the inhibitor (Equation 19).

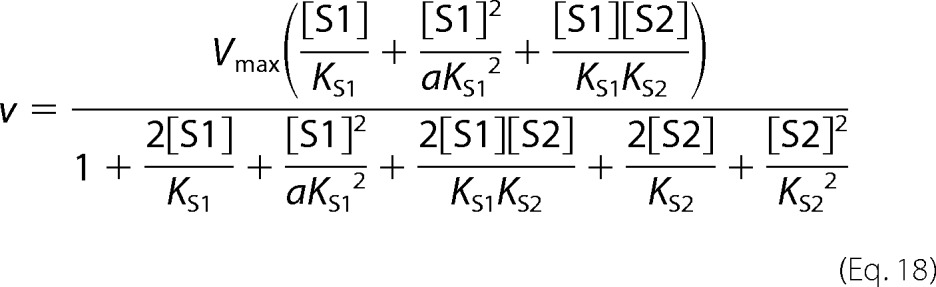


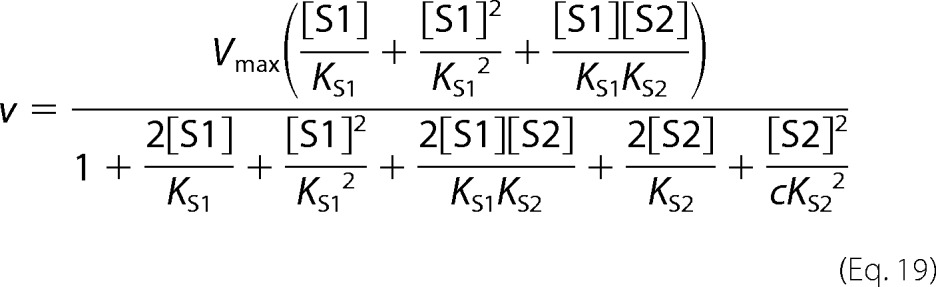
 Two-site pure competitive inhibition is given by Equation 20.

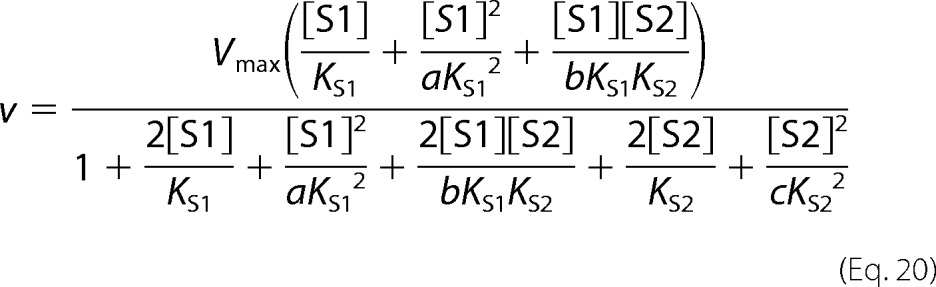
 Partial competitive inhibition is given by Equation 21.

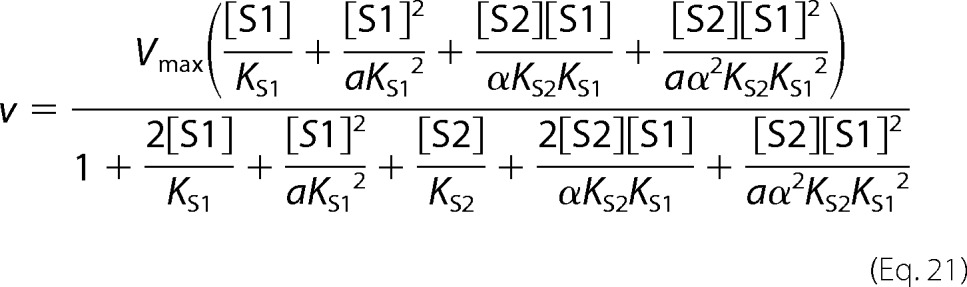
 For substrate non-cooperative in the absence of inhibitor, the substrate reverses the effect of the inhibitor.

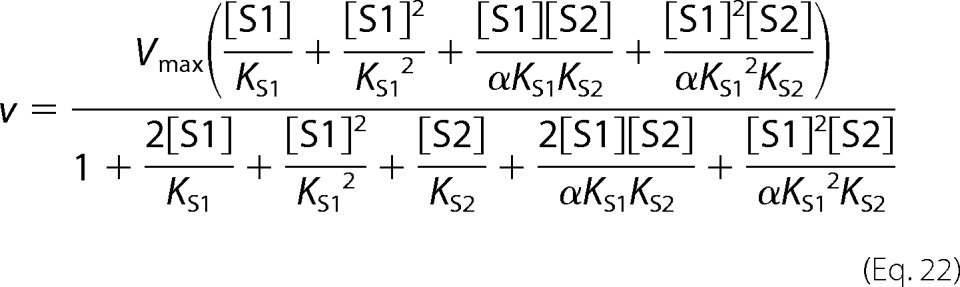
 For substrate cooperative in the absence of the inhibitor, the substrate reverses the effect of the inhibitor.

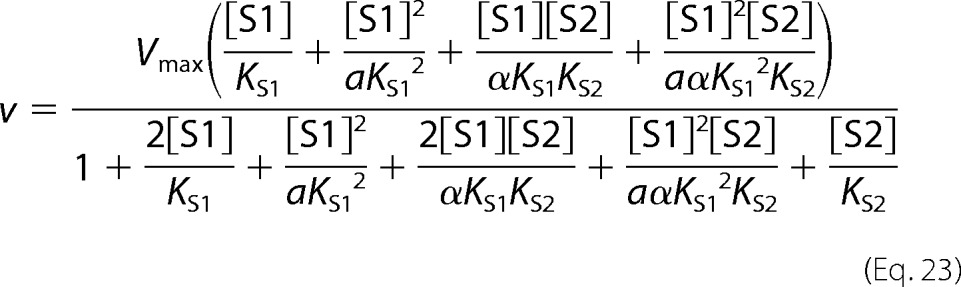
 Inhibitor eliminates substrate cooperativity.

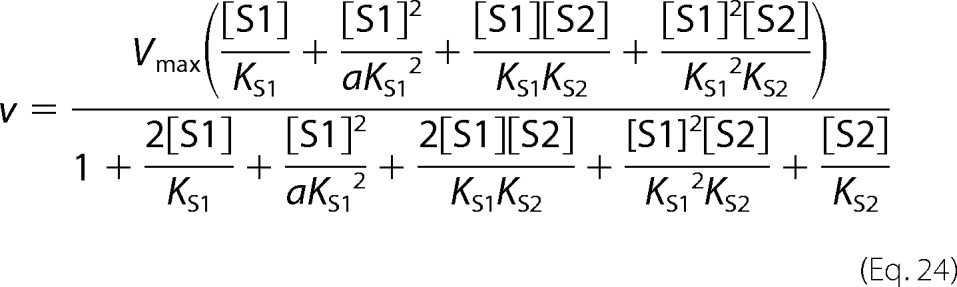


##### Competition Plot

The competition plot was performed as described elsewhere (35). Briefly, the substrate with the lower *V*_max_ was termed “A” (in this case quinine), and the substrate with the higher *V*_max_ was termed “B” (in this case chloroquine). A concentration of chloroquine (from the 0 μm quinine treatment of Fig. 2*A*) and a concentration of quinine (from the 0 μm chloroquine treatment of Fig. 4*A*) were selected, such that (i) the rate of chloroquine transport was equivalent to the rate of quinine transport, and (ii) the transport rate for quinine (which was the less efficiently transported substrate) approached its *V*_max_ (9.3 ± 0.2 pmol of quinine/h/oocyte). The resulting concentrations were *a*_0_ = 73.5 μm and *b*_0_ = 52.1 μm for quinine and chloroquine, respectively. At these concentrations of quinine and chloroquine, *v_a_* = *v_b_* = 7.4 pmol/h/oocyte. A series of reaction buffers containing A and B at concentrations *a* = (1 − *P*) × *a*_0_ and *b* = *P* × b_0_ were assembled (with 0 ≤ *P* ≤ 1). For each of these mixtures, the individual velocities *v_a_* and *v_b_*, as well as with the total rate of drug transport (*V*_total_ = *v_a_* + *v_b_*), were predicted from the kinetic data presented in Figs. 2*A* and 4*A*. The resulting values are presented in Table 1. The rates of chloroquine and quinine transport were then measured in pairwise experiments by the addition of either [^3^H]chloroquine or [^3^H]quinine to each of the unlabeled chloroquine/quinine mixtures (boldface type in Table 1). These corresponded to *P* values of 0, 0.15, 0.4, 0.6, 0.9, and 1.0. A plot of *P* as a function of *V*_total_ yielded the competition plot.

**TABLE 1:** **Predicted rates of quinine, chloroquine, and total drug transport in the presence of different concentrations of quinine and chloroquine** Quinine is denoted as substrate A and chloroquine as substrate B. *P* is the proportion of quinine in the chloroquine/quinine mixture, *v_a_* and *v_b_*, are the respective rates of quinine and chloroquine transport, and *V*_total_ is the sum of *v_a_* and *v_b_*. The values for *v_a_* and *v_b_* were calculated from the kinetic data presented in Figs. 2*A* and 4*A*.

*P*	*v_a_*	*v_b_*	*V*_total_	[Quinine]	[Chloroquine]
	*pmol/h/oocyte*	*pmol/h/oocyte*	*pmol/h/oocyte*	μ*m*	μ*m*
**0.00**	**7.40**	**0.00**	**7.40**	**73.50**	**0.00**
0.05	5.79	0.11	5.90	69.83	2.61
0.10	5.67	0.24	5.91	66.15	5.21
**0.15**	**5.54**	**0.37**	**5.91**	**62.48**	**7.82**
0.20	5.41	0.51	5.91	58.80	10.42
0.25	5.26	0.66	5.92	55.13	13.03
0.30	5.11	0.82	5.93	51.45	15.63
0.35	4.94	1.00	5.94	47.78	18.24
**0.40**	**4.76**	**1.19**	**5.95**	**44.10**	**20.84**
0.45	4.56	1.40	5.96	40.43	23.45
0.50	4.34	1.64	5.98	36.75	26.05
0.55	4.11	1.91	6.02	33.08	28.66
**0.60**	**3.84**	**2.20**	**6.05**	**29.40**	**31.26**
0.65	3.54	2.54	6.09	25.73	33.87
0.70	3.21	2.92	6.13	22.05	36.47
0.75	2.80	3.37	6.17	18.38	39.08
0.80	2.34	3.90	6.24	14.70	41.68
0.85	1.84	4.53	6.37	11.03	44.29
**0.90**	**1.29**	**5.30**	**6.60**	**7.35**	**46.89**
0.95	0.69	6.25	6.94	3.68	49.50
**1.00**	**0.00**	**7.40**	**7.40**	**0.00**	**52.10**

## RESULTS

### 

#### 

##### PfCRT^Dd2^ Mediates the Saturable Transport of Quinine and Quinidine

**FIGURE 1.**
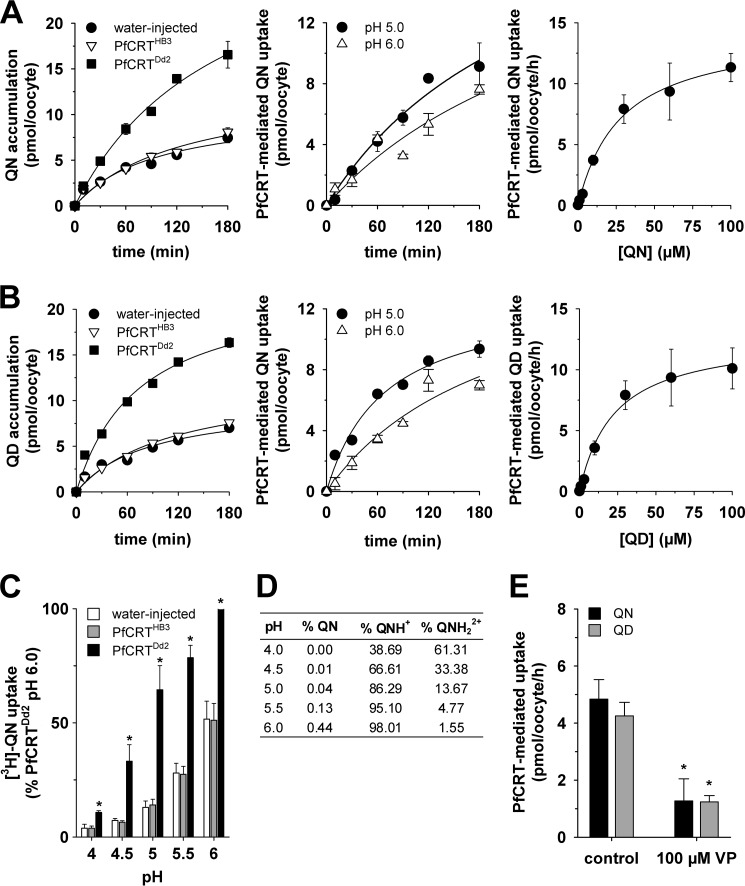
**The transport of quinine and quinidine into *Xenopus* oocytes expressing PfCRT.** *A* and *B*, PfCRT^Dd2^ mediates quinine (*QN*) and quinidine (*QD*) uptake, respectively. *Left panels*, time courses for the uptake of quinine and quinidine into water-injected oocytes (*solid circles*) and oocytes expressing PfCRT^HB3^ (*triangles*) or PfCRT^Dd2^ (*solid squares*). The extracellular pH was 5.0, and the total concentration of the drug (radiolabeled plus unlabeled) was 10 μm. *Middle panels*, PfCRT^Dd2^-mediated uptake of quinine or quinidine at pH 5.0 and 6.0. The PfCRT^Dd2^-mediated component of transport was obtained by subtracting uptake in oocytes expressing PfCRT^HB3^ from that measured in PfCRT^Dd2^-expressing oocytes. *Right panels*, concentration dependence of the PfCRT^Dd2^-mediated uptake of quinine and quinidine. In both cases, the uptake of radiolabeled drug into water-injected oocytes and oocytes expressing PfCRT^Dd2^ was measured at pH 6.0 over an extracellular concentration range of 0.05–100 μm drug (radiolabeled plus unlabeled). The rate of PfCRT^Dd2^-mediated transport was calculated by subtracting the rate measured in water-injected oocytes from that in oocytes expressing PfCRT^Dd2^ at each quinine or quinidine concentration. A least-squares fit of the Michaelis-Menten equation to the resulting data (using Sigma Plot version 12.5) yielded the following kinetic parameters: quinine, apparent *K_m_* = 28 ± 4 μm and apparent *V*_max_ = 14 ± 1 pmol/h/oocyte; quinidine, *K_m_* = 23 ± 4 μm and apparent *V*_max_ = 13 ± 1 pmol/h/oocyte. *C*, pH dependence of quinine uptake into water-injected oocytes and oocytes expressing PfCRT^HB3^ or PfCRT^Dd2^. Measurements of radiolabeled quinine transport were made over the extracellular pH range (pH 4.0–6.0) and in the presence of 1 μm unlabeled quinine. *Asterisks* indicate significant differences in the accumulation of quinine between the control (water-injected or PfCRT^HB3^-expressing) oocytes and oocytes expressing PfCRT^Dd2^ within each pH condition (*, *p* < 0.05). *D*, percentages of quinine in the neutral (*QN*), monoprotonated (*QN*^+^), and diprotonated (*QN^2^*^+^) forms in solutions of different pH. The percentages were calculated using the Henderson-Hasselbalch equation, with p*K_a_* values of 4.12 for the quinoline nitrogen and 8.58 for the side chain nitrogen (51). *E*, the effect of verapamil (100 μm) on the PfCRT^Dd2^-mediated transport of quinine (*black bars*) and quinidine (*gray bars*) was measured at pH 5.0. The total extracellular concentration of quinine or quinidine (radiolabeled plus unlabeled) was 10 μm. *Asterisks* indicate significant differences in the PfCRT^Dd2^-mediated uptake of radiolabeled drug between the control oocytes and those suspended in 100 μm verapamil (*, *p* < 0.05). In all cases, uptake is shown as the mean ± S.E. (*error bars*) of at least three biological repeats, within which measurements were made from 10 oocytes/treatment.

Quinine and its stereoisomer quinidine are diprotic weak bases with p*K_a_* values of 4.12 and 8.58 and 4.42 and 8.58, respectively (where *K_a_* is the acid dissociation constant). Both drugs can permeate membranes as free bases and will partition between membrane-bound compartments according to the prevailing pH gradient. The uptake of radiolabeled quinine and quinidine was measured in an acidic medium (pH 5.0) into *Xenopus* oocytes expressing either PfCRT^Dd2^ or the wild-type protein from the HB3 strain (PfCRT^HB3^) as well as into water-injected oocytes. Oocytes expressing PfCRT^Dd2^ took up significantly more quinine and quinidine relative to water-injected oocytes or oocytes expressing PfCRT^HB3^ (*left panels* of Fig. 1, *A* and *B*, respectively). Similar results were obtained when the time courses were performed at pH 6.0 (data not shown). The level of uptake in oocytes expressing PfCRT^HB3^ was not significantly different from that measured in water-injected oocytes, indicating that the accumulation of quinine and quinidine in these oocytes and in the water-injected oocytes was most likely due to the simple diffusion of the uncharged forms of these drugs. Consistent with this observation, quinine transport was shown to be dependent on the pH of the medium (Fig. 1*C*). That is, the accumulation of quinine in the control oocytes increased as the pH was raised from 4.0 to 6.0, which correlated well with the pH dependence of the concentration of the uncharged quinine present in the medium (Fig. 1*D*). Nevertheless, under each of the conditions tested, the accumulation of quinine in oocytes expressing PfCRT^Dd2^ was significantly higher than that measured in water-injected oocytes and in oocytes expressing PfCRT^HB3^ (*p* < 0.05).

The portion of quinine or quinidine accumulation attributable to PfCRT^Dd2^ was calculated by subtracting the uptake in oocytes expressing PfCRT^HB3^ from that measured in PfCRT^Dd2^-expressing oocytes (*middle panels* of Fig. 1, *A* and *B*, respectively). The resulting data indicated that the PfCRT^Dd2^-mediated uptake of quinine and quinidine was approximately linear with time for at least 60 min at both pH 5.0 and pH 6.0.

We also tested the ability of the resistance reverser verapamil to inhibit the uptake of quinine and quinidine via PfCRT^Dd2^ at pH 5.0 (Fig. 1*E*). Verapamil has previously been shown to inhibit chloroquine transport via PfCRT^Dd2^ (24), and consistent with this activity, the addition of 100 μm verapamil significantly reduced the PfCRT^Dd2^-mediated uptake of quinine and quinidine.

Given that the time dependence of PfCRT^Dd2^-mediated uptake at pH 6.0 was roughly comparable to that obtained at pH 5.0, and in order to facilitate comparisons with previous kinetic analyses of transport via PfCRT^Dd2^ (which were performed at pH 6.0 (24, 25)), the saturability of quinine and quinidine transport was determined at pH 6.0. For both quinine and quinidine, the uptake of radiolabeled drug in PfCRT^Dd2^-expressing oocytes decreased with increasing concentrations of the unlabeled drug, consistent with transport occurring via a saturable mechanism (*right panels* of Fig. 1, *A* and *B*, respectively). A least squares fit of the Michaelis-Menten equation to the data yielded apparent Michaelis constants (*K_m_*) of 28 ± 4 and 23 ± 4 μm and maximal velocities (*V*_max_) of 14 ± 1 and 13 ± 1 pmol/h/oocyte for quinine and quinidine, respectively.

##### Chloroquine and Quinine are Mixed-type Inhibitors of PfCRT^Dd2^

**FIGURE 2.**
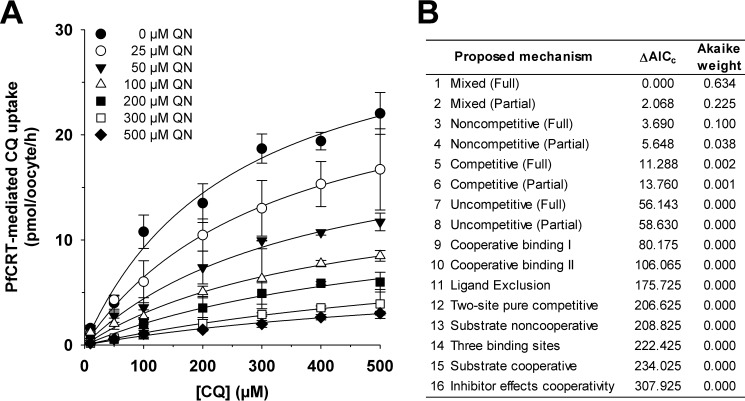
**Kinetic analysis of the inhibition of PfCRT^Dd2^-mediated chloroquine transport by quinine.** *A*, the uptake of chloroquine (*CQ*) into water-injected oocytes and oocytes expressing PfCRT^Dd2^ was measured at pH 6.0 and in the presence of a total extracellular chloroquine concentration (radiolabeled plus unlabeled) of 10, 50, 100, 200, 300, 400, or 500 μm. At each of these chloroquine concentrations, the effects of seven concentrations of unlabeled quinine (*QN*; ranging from 0 to 500 μm) were tested. For each of the 49 treatments, the rate of chloroquine transport attributable to PfCRT^Dd2^ was then calculated by subtracting the rate measured in water-injected oocytes from that in oocytes expressing PfCRT^Dd2^. Chloroquine uptake is shown as the mean ± S.E. (*error bars*) of at least three biological repeats, within which measurements were made from 10 oocytes/treatment. *B*, Sixteen different models of inhibition were globally fitted to the data presented in *A* using the least-squares method. The plausibility of each model was evaluated by calculating the Akaike information criterion difference (Δ*AIC_c_*) and the Akaike weight (33, 34). The *table* shows the models in descending order (*i.e.* the most plausible model is listed first). The kinetic parameters derived from the two most plausible inhibition models are presented in Table 2.

The finding that PfCRT^Dd2^ transports both quinine and chloroquine led us to undertake a series of kinetic analyses to assess the mechanism by which PfCRT accepts different substrates. Substrate competition experiments were conducted in which the uptake of radiolabeled chloroquine was determined at 60 min (*i.e.* within the initial phase of chloroquine uptake (24)) in water-injected oocytes and oocytes expressing PfCRT^Dd2^. In these assays, the extracellular medium contained one of seven concentrations of unlabeled chloroquine (ranging from 10 to 500 μm), and at each of these chloroquine concentrations, the effects of seven concentrations of unlabeled quinine (ranging from 0 to 500 μm) were tested. The rate of PfCRT-mediated chloroquine uptake was calculated, and each of the seven quinine data sets was plotted as a function of the chloroquine concentration (Fig. 2*A*). To determine the nature of the interaction between PfCRT, chloroquine, and quinine, we performed a least-squares global fit of 16 different models of inhibition (32) to the data. The inhibition models were then ranked according to two measures that reflect how well a given model explains the experimentally derived data: the second-order Akaike information criterion difference (Δ*AIC_c_*) and the Akaike weight (33) (Fig. 2*B*). The resulting rankings were assessed using the heuristic criteria devised by Burnham and Anderson (33), in which models with Δ*AIC_c_* < 2 are equally plausible, models with 4 < Δ*AIC_c_* < 7 are considerably less likely, and models with Δ*AIC_c_* > 10 are considered unlikely. The Akaike weight varies from 1 to 0, with the plausibility of the model increasing as the Akaike weight approaches 1. According to these analyses, the most plausible models for the binding of quinine to PfCRT were full mixed-type inhibition followed by partial mixed-type inhibition (Fig. 2*B* and Table 2). The respective values for Δ*AIC_c_* (0 and 2.0) suggested that both models were equally credible. We therefore applied an F-test to discriminate between full mixed-type inhibition and partial mixed-type inhibition, the results of which indicated that the former model provided the better fit to the data (*F* = 0.5; *p* < 0.01).

**TABLE 2:** **Kinetic parameters describing interactions of PfCRT^Dd2^ with chloroquine, quinine, and verapamil** Shown are the best-fit values of kinetic parameters obtained using the full and partial mixed-type inhibition models. The drug present in radiolabeled form was denoted as the substrate, and the unlabeled drug was denoted as the inhibitor. CI, confidence interval; *V*_max_, the maximum velocity of substrate transport; *K*_S_^CQ^, *K*_S_^QN^, and *K*_S_^VP^, the dissociation constants for the chloroquine-PfCRT^Dd2^, quinine-PfCRT^Dd2^, and verapamil-PfCRT^Dd2^ complexes, respectively; α, the factor by which these *K*_S_ values change when the opposing substrate is already bound to the transporter; β, the factor by which the *V*_max_ is affected by the inhibitor; CQ, chloroquine; QN, quinine; VP, verapamil.

Substrate	Inhibitor	Parameters	Mixed (full)		Mixed (partial)	
			Mean ± S.E.	95% CI	Mean ± S.E.	95% CI
CQ	QN	*V*_max_ (pmol/oocyte/h)	33 ± 2	29–37	33 ± 2	29–37
		*K*_S_^CQ^ (μm)	250 ± 30	192–315	250 ± 30	192–316
		*K*_S_^QN^ (μm)	40 ± 7	26–54	40 ± 7	25–53
		α	2.6 ± 1	0.3–5	2.6 ± 1	0.3–5
		β			0.02 ± 0.03	−0.04–0.1
QN	CQ	*V*_max_ (pmol/oocyte/h)	9 ± 0.2	9–10	9 ± 0.2	9–10
		*K*_S_^QN^ (μm)	25 ± 1	22–28	25 ± 1	22–28
		*K*_S_^CQ^ (μm)	340 ± 50	240–440	342 ± 54	234–450
		α	1.9 ± 0.5	0.9–2.8	2 ± 0.5	0.9–2.8
		β			0.00 ± 0.03	−0.05–0.05
CQ	VP	*V*_max_ (pmol/oocyte/h)	27 ± 0.3	26–27	27 ± 0.3	26–27
		*K*_S_^CQ^ (μm)	195 ± 6	183–208	196 ± 6	185–207
		*K*_S_^VP^ (μm)	36 ± 2	32–40	35 ± 2	31–38
		α	2.9 ± 0.4	2–4	3 ± 0.3	2–3
		β			0.03 ± 0.01	0.01–0.05

The models ranked third and fourth were full and partial noncompetitive inhibition, respectively. Although the Akaike weights for these two models were low, the Δ*AIC_c_* values (which were between 3 and 6) indicated that neither model could be immediately dismissed. However, cases of noncompetitive inhibition are rare in nature and appear to be primarily restricted to small inhibitors, such as protons or other inorganic ions (36). Given that chloroquine and quinine are large organic compounds (both have molecular weights greater than 300 g/mol), it is unlikely that quinine is a noncompetitive inhibitor of chloroquine transport via PfCRT.

**FIGURE 3.**
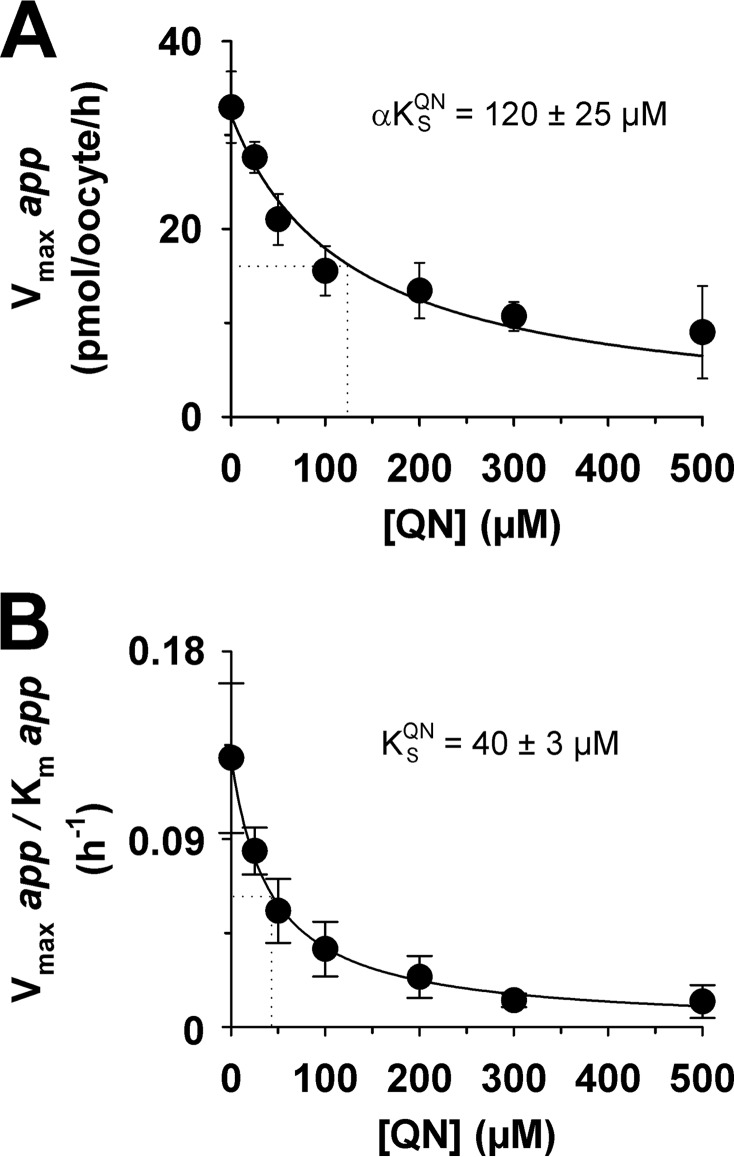
**Secondary analyses of the inhibition of PfCRT^Dd2^-mediated chloroquine transport by quinine.** The Michaelis-Menten equation was fitted to the kinetic data shown in Fig. 2*A* to derive the apparent *K_m_* and apparent *V*_max_ values for chloroquine transport at each of the seven concentrations of quinine (*QN*). *A*, The resulting apparent *V*_max_ values were plotted as a function of the quinine concentration and a rectangular hyperbolic equation fitted to the data. The *dotted line* indicates the quinine concentration at which the *V*_max_ for chloroquine transport was half-maximal. This value is the dissociation constant for the binding of quinine to the chloroquine-PfCRT^Dd2^ complex (α*K*_S_^QN^). *B*, the ratio of the apparent *V*_max_ to its corresponding apparent *K_m_* was plotted as a function of the quinine concentration and a rectangular hyperbolic equation fitted to the data. The *dotted line* indicates the quinine concentration at which the *V*_max_/*K_m_* ratio was half-maximal. This value equates to the dissociation constant for the binding of quinine to the empty transporter (*K*_S_^QN^).

Mixed-type inhibition occurs when an inhibitor can bind to an enzyme (or, in this case, a transporter) regardless of whether the substrate-binding site is occupied or empty but exhibits greater potency against one binding state over the other. Mixed-type inhibitors affect the apparent affinity of the transporter for the substrate (*i.e.* the *K_m_*) and also cause a decrease in the apparent maximum rate of transport (*i.e.* the *V*_max_). Hence, if quinine behaves as a mixed-type inhibitor of chloroquine transport, we would expect it to affect both the apparent *K_m_* and apparent *V*_max_ of chloroquine transport via PfCRT^Dd2^. To test this possibility, we reanalyzed the data presented in Fig. 2*A* by fitting the Michaelis-Menten equation to each of the seven data sets (which differed in the concentration of quinine that was present). The resulting apparent *V*_max_ values as well as the ratios of *V*_max_ to *K_m_* were plotted as a function of the quinine concentration (Fig. 3, *A* and *B*, respectively). Both of these values decreased with increasing concentrations of unlabeled quinine, resulting in hyperbolic curves. The half-maximum inhibitory concentration derived from Fig. 3*A*, which equates to the dissociation constant for the binding of quinine to the chloroquine-PfCRT^Dd2^ complex (α*K*_S_ (36)), was 120 ± 25 μm. By contrast, the half-maximum inhibitory concentration derived from Fig. 3*B* (40 ± 3 μm) gives the dissociation constant for the binding of quinine to the empty transporter (*K*_S_). Taken together, these data indicate that (i) the presence of chloroquine in the substrate-binding site reduces the transporter's affinity for quinine by a factor (α) of ∼3, and (ii) although quinine's inhibition of chloroquine transport is mostly a competitive process, there is also an uncompetitive component (Table 2).

**FIGURE 4.**
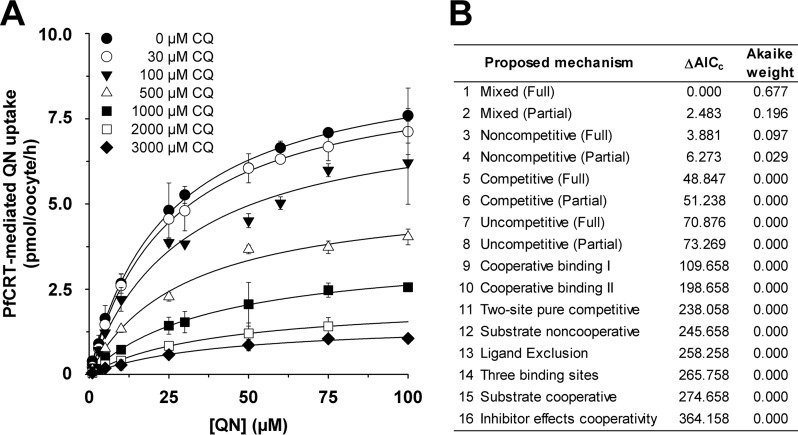
**Kinetic analysis of the inhibition of PfCRT^Dd2^-mediated quinine transport by chloroquine.** *A*, the uptake of quinine (*QN*) into water-injected oocytes and oocytes expressing PfCRT^Dd2^ was measured at pH 6.0 and in the presence of a total extracellular quinine concentration (radiolabeled plus unlabeled) of 1, 3, 5, 10, 25, 30, 50, 60, 75, or 100 μm. At each of these quinine concentrations, the effects of seven concentrations of unlabeled chloroquine (*CQ*; ranging from 0 to 3 mm) were tested. For each of the 70 treatments, the rate of quinine transport attributable to PfCRT^Dd2^ was then calculated by subtracting the rate measured in water-injected oocytes from that in oocytes expressing PfCRT^Dd2^. Quinine uptake is shown as the mean ± S.E. (*error bars*) of at least four biological repeats, within which measurements were made from 10 oocytes/treatment. *B*, Sixteen different models of inhibition were globally fitted to the data presented in *A* using the least-squares method. The plausibility of each model was evaluated by calculating the Akaike information criterion difference (Δ*AIC_c_*) and the Akaike weight (33, 34). The *table* shows the models in descending order (*i.e.* the most plausible model is listed first). Kinetic parameters derived from the two most plausible inhibition models are presented in Table 2.

**FIGURE 5.**
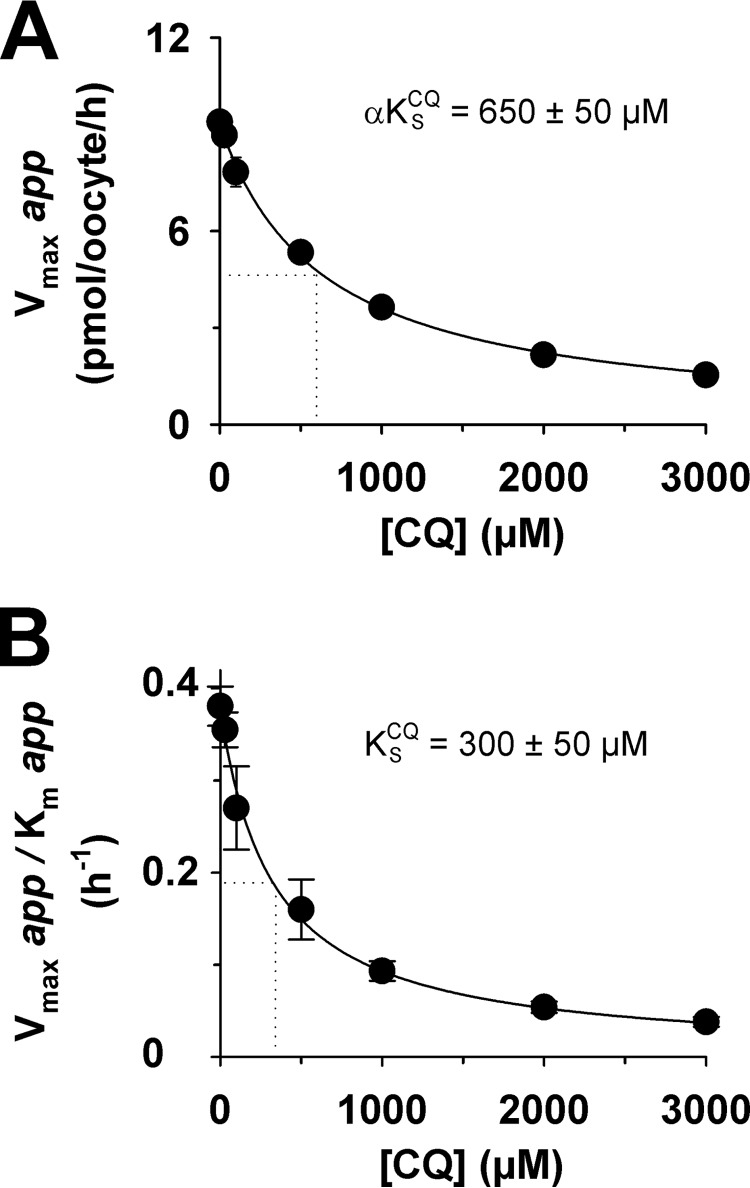
**Secondary analyses of the inhibition of PfCRT^Dd2^-mediated quinine transport by chloroquine.** The Michaelis-Menten equation was fitted to the kinetic data shown in Fig. 4*A* to derive the apparent *K_m_* and apparent *V*_max_ values for quinine transport at each of the seven concentrations of chloroquine (*CQ*). *A*, the resulting apparent *V*_max_ values were plotted as a function of the chloroquine concentration and a rectangular hyperbolic equation fitted to the data. The *dotted line* indicates the chloroquine concentration at which the *V*_max_ for quinine transport was half-maximal. This value is the dissociation constant for the binding of chloroquine to the quinine-PfCRT^Dd2^ complex (α*K*_S_^CQ^). *B*, the ratio of the apparent *V*_max_ to its corresponding apparent *K_m_* was plotted as a function of the chloroquine concentration and a rectangular hyperbolic equation fitted to the data. The *dotted line* indicates the chloroquine concentration at which the *V*_max_/*K_m_* ratio was half-maximal. This value equates to the dissociation constant for the binding of chloroquine to the empty transporter (*K*_S_^CQ^). *Error bars*, S.E.

A complementary set of experiments was undertaken to investigate the ability of chloroquine to inhibit quinine transport via PfCRT^Dd2^. In assays similar to those described above, the uptake of radiolabeled quinine was measured in the presence of different concentrations of unlabeled quinine (ranging from 0 to 100 μm), and at each of these quinine concentrations, the effects of seven concentrations of unlabeled chloroquine (ranging from 0 to 3 mm) were measured. The 16 models of inhibition were fitted to the resulting data (presented in Fig. 4*A*) and ranked according to Δ*AIC_c_* and the Akaike weight (Fig. 4*B*). These analyses suggested that the most plausible models were again full mixed-type inhibition and partial mixed-type inhibition (Δ*AIC_c_* of 0 and 2.5, respectively; Fig. 4*B*), with an F-test indicating that full mixed-type inhibition best described the data (*F* = 0; *p* < 10^−15^). Moreover, increasing the concentration of unlabeled chloroquine decreased the apparent *V*_max_ of quinine transport (Fig. 5*A*) and increased the apparent *K_m_* of PfCRT^Dd2^ for quinine (Fig. 5*B*). The corresponding α*K*_S_ and *K*_S_ values were 650 ± 50 and 300 ± 50 μm, respectively. These findings further support the idea that the affinity of PfCRT^Dd2^ for one drug decreases when the carrier is occupied by a second drug.

**FIGURE 6.**
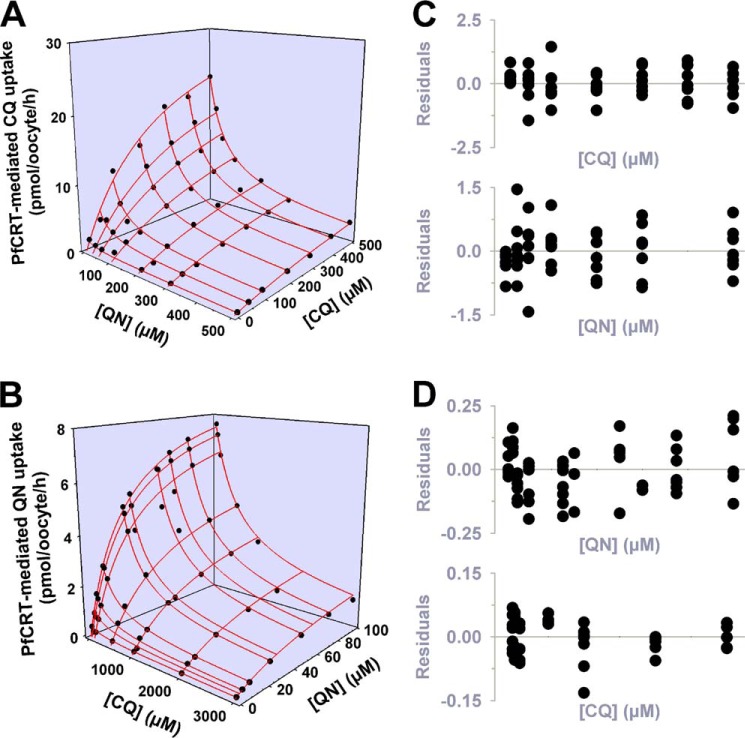
**Modeling the interaction between PfCRT^Dd2^, chloroquine, and quinine using the full mixed-type inhibition equation.** To test how well the full mixed-type inhibition model fitted the kinetic data presented in Figs. 2*A* and 4*A*, the relevant equation was solved using the kinetic parameters for the transport of quinine (*QN*) and chloroquine (*CQ*) via PfCRT^Dd2^ as well as of their inhibition of the transporter. The resulting predicted values (*red lines*) were then displayed as a three-dimensional plot, with the experimentally derived data shown for comparison. *A*, inhibition of chloroquine transport by quinine. The following values were used: *V*_max_ = 33 pmol of chloroquine/h/oocyte; chloroquine concentrations = 0–500 μm; chloroquine *K*_S_^CQ^ = 270 μm; quinine *K*_S_^QN^ = 32 μm; quinine concentrations = 0–500 μm; α = 2.5. *B*, inhibition of quinine transport by chloroquine. The following values were used: *V*_max_ = 9.6 pmol of quinine/h/oocyte; quinine concentration = 0–100 μm; quinine *K*_S_^QN^ = 32 μm; chloroquine *K*_S_^CQ^ = 270 μm; chloroquine concentrations = 0–3000 μm; α = 2.5. *C* and *D*, in accordance with the analysis described by Cornish-Bowden (37), the difference between the experimentally derived data and the predicted values was calculated, and the resulting residuals are displayed as a function of (i) the chloroquine concentration and (ii) the quinine concentration.

Having obtained complementary sets of kinetic parameters for the transport of quinine and chloroquine via PfCRT^Dd2^, as well as of their inhibition of the transporter, we modeled the kinetics of the interaction between PfCRT^Dd2^, chloroquine and quinine using the full mixed-type inhibition equation. Three-dimensional plots of the modeled system are presented in Fig. 6, *A* and *B*, with the experimentally derived data (from Fig. 2*A* or 4*A*, respectively) superimposed. To assess how well the model fitted the data, we calculated the difference between the observed and expected uptake values and plotted the resulting residual values as a function of quinine or chloroquine concentration (Fig. 6, *C* and *D*, respectively) (37). Within each concentration of chloroquine or quinine, the residuals did not tend to cluster to one side or the other of the *x* axis but were instead randomly distributed across the *x* axis. This pattern indicates that the model is an appropriate fit to the experimentally derived data, thereby providing further evidence that full mixed-type inhibition is a plausible model for the interaction of PfCRT^Dd2^ with chloroquine and quinine.

##### Distinct Binding Sites for Chloroquine and Quinine

**FIGURE 7.**
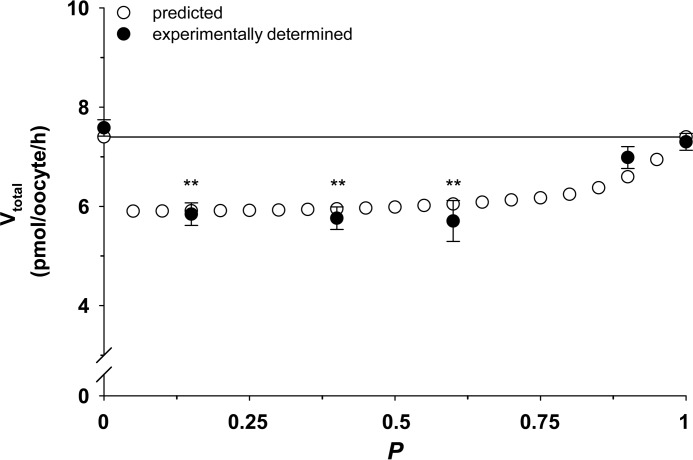
**Competition plot of the interaction of PfCRT^Dd2^ with chloroquine and quinine.** The competition plot tests whether two reactions occur at the same site or at distinct sites (35). An extracellular concentration of quinine (73.5 μm) was selected from the data presented in Fig. 4*A*, such that the resulting rate of quinine transport (7.4 pmol/h/oocyte) would approach its *V*_max_. The concentration of chloroquine (52.1 μm) that would result in the equivalent rate of chloroquine transport was estimated from the data shown in Fig. 2*A*. Pairwise measurements of radiolabeled chloroquine and radiolabeled quinine were made in the presence of different proportions of these chloroquine and quinine concentrations (see “Experimental Procedures”), from which the total rate of drug transport within each of the chloroquine/quinine mixtures was calculated. The resulting total PfCRT^Dd2^-mediated transport velocities (*V*_total_) were plotted as a function of the proportion of quinine in the chloroquine/quinine mixture (*P*). The total rate of drug uptake (*closed circles*) is shown as the mean ± S.E. (*error bars*) of four biological repeats, within which measurements were made from 10 oocytes/treatment. If the two drugs compete for binding to the same site, the total rate of drug transport within each of the chloroquine/quinine mixtures would be ∼7.4 pmol/h/oocyte (indicated by the *solid line*). By contrast, if chloroquine and quinine bind to distinct but antagonistically interacting sites, the total rate of drug transport will vary with *P*, resulting in a concave curve (*open circles*; values were predicted as described under “Experimental Procedures” using a full mixed-type inhibition model). Those experiments in which *P* equaled 0.15, 0.40, 0.60, or 0.9 yielded rates that were significantly different from 7.4 pmol/h/oocyte (*p* < 0.01; one-way analysis of variance).

Many enzymes and some transporters bind substrates by induced fit, whereby the binding of a substrate alters the conformation of the protein (32, 38). If PfCRT functions according to an induced fit mechanism, different substrates could induce distinct conformational changes, and even if the substrates compete for binding at the same site, the differences between these conformations may account for the mixed-type mechanism of inhibition identified here for chloroquine and quinine. To test whether chloroquine and quinine bind to the same site or at distinct sites, we performed the competition plot experiment (35). To this end, we selected a concentration of chloroquine (from the 0 μm quinine treatment of Fig. 2*A*) and a quinine concentration (from the 0 μm chloroquine treatment of Fig. 4*A*) that gave the same rate of drug transport (35). Measurements of radiolabeled chloroquine or radiolabeled quinine were then made in the presence of different proportions of these chloroquine and quinine concentrations (calculated using the equations set out under “Experimental Procedures”). For each of the resulting chloroquine/quinine mixtures, the rate of PfCRT-mediated chloroquine and quinine transport was determined (in fluxes performed pairwise) and then added together to yield a “total” rate of drug transport. These rates were plotted as a function of the proportion of quinine in the chloroquine/quinine mixture (*P*). If two substrates bind to the same site, the total rate of drug transport will be independent of *P*. However, if the substrates bind to different but antagonistically interacting sites, the total rate of drug transport will vary with *P*, resulting in a concave curve (35). Given that the latter relationship was apparent for PfCRT^Dd2^ (Fig. 7), it is unlikely that the mixed-type inhibition kinetics observed for chloroquine and quinine arise from a special case of product inhibition. This finding, together with the analyses of the kinetics of inhibition, suggests that there are distinct but interdependent binding sites within PfCRT^Dd2^ for chloroquine and quinine.

##### Verapamil Acts as a Partial Mixed-type Inhibitor of PfCRT^Dd2^-mediated Chloroquine Transport

**FIGURE 8.**
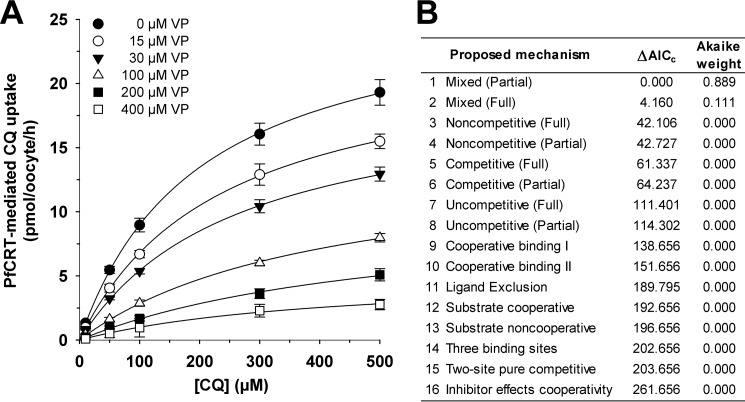
**Kinetic analysis of the inhibition of PfCRT^Dd2^-mediated chloroquine transport by verapamil.** *A*, the uptake of chloroquine (*CQ*) into water-injected oocytes and oocytes expressing PfCRT^Dd2^ was measured at pH 6.0 and in the presence of a total extracellular chloroquine concentration (radiolabeled plus unlabeled) of 10, 50, 100, 300, or 500 μm. At each of these chloroquine concentrations, the effects of six concentrations of unlabeled verapamil (*VP*; ranging from 0 to 400 μm) were tested. For each of the 30 treatments, the rate of chloroquine transport attributable to PfCRT^Dd2^ was then calculated by subtracting the rate measured in water-injected oocytes from that in oocytes expressing PfCRT^Dd2^. Chloroquine uptake is shown as the mean ± S.E. (*error bars*) of at least eight biological repeats, within which measurements were made from 10 oocytes/treatment. *B*, Sixteen different models of inhibition were globally fitted to the data presented in *A* using the least-squares method. The plausibility of each model was evaluated by calculating the Akaike information criterion difference (Δ*AIC_c_*) and the Akaike weight (33, 34). The *table* shows the models in descending order (*i.e.* the most plausible model is listed first). The kinetic parameters derived from the two most plausible inhibition models are presented in Table 2.

**FIGURE 9.**
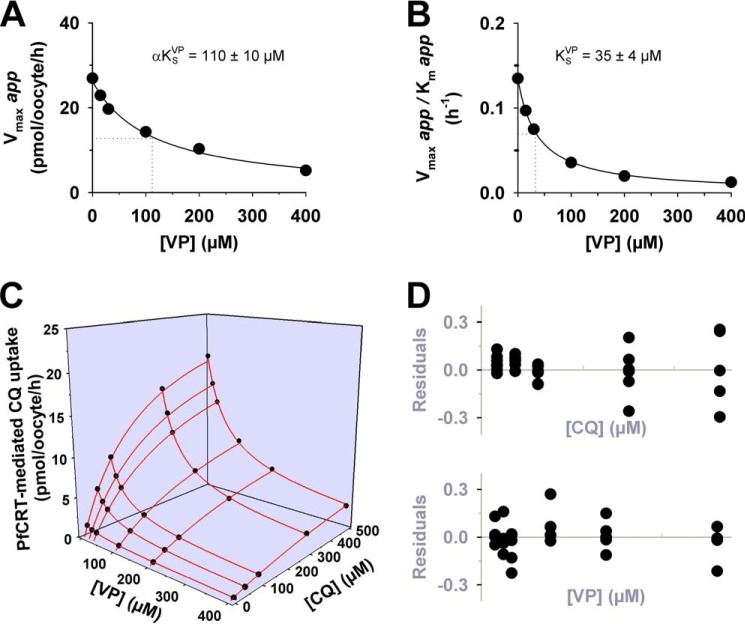
**Secondary analyses and modeling of the inhibition of PfCRT^Dd2^-mediated chloroquine transport by verapamil.** *A*, the Michaelis-Menten equation was fitted to the kinetic data shown in Fig. 8 to derive the apparent *K_m_* and apparent *V*_max_ values for chloroquine (*CQ*) transport at each of the six concentrations of verapamil (*VP*). The resulting apparent *V*_max_ values were plotted as a function of the verapamil concentration and a rectangular hyperbolic equation fitted to the data. The *dotted line* indicates the verapamil concentration at which the *V*_max_ for chloroquine transport was half-maximal. This value is the dissociation constant for the binding of verapamil to the chloroquine-PfCRT^Dd2^ complex (α*K*_s_^VP^). *B*, the ratio of the apparent *V*_max_ to its corresponding apparent *K_m_* was plotted as a function of the verapamil concentration and a rectangular hyperbolic equation fitted to the data. The *dotted line* indicates the verapamil concentration at which the *V*_max_/*K_m_* ratio was half-maximal. This value equates to the dissociation constant for the binding of verapamil to the empty transporter (*K*_s_^VP^). *C*, the partial mixed-type inhibition equation was solved using the following values: *V*_max_ = 26 pmol of chloroquine/h/oocyte; chloroquine concentrations = 0–500 μm; chloroquine *K*_S_ = 270 μm; verapamil *K*_s_^VP^ = 36 μm; verapamil concentrations = 0–400 μm; α = 2.5; β = 0.03. The resulting predicted values (*red lines*) were then displayed as a three-dimensional plot, with the experimentally derived data shown for comparison. *D*, in accordance with the analysis described by Cornish-Bowden and co-workers (35), the difference between the experimentally derived data and the predicted values was calculated, and the resulting residuals were displayed as a function of (i) the chloroquine concentration and (ii) the verapamil concentration.

Verapamil is an inhibitor of PfCRT^Dd2^ and a reverser of chloroquine resistance *in vitro* (7, 24, 30). At present, the clinical application of resistance reversers (such as verapamil) has been prevented by problems with potency and host toxicity. Little is known about the mechanism by which verapamil inhibits PfCRT^Dd2^, yet it is possible that an understanding of this interaction could lead to the development of more potent inhibitors of the transporter. We investigated the interaction between verapamil and PfCRT^Dd2^ by conducting substrate competition experiments similar to those described for quinine and chloroquine. The PfCRT-mediated uptake of radiolabeled chloroquine was measured at five different concentrations of unlabeled chloroquine (0, 50, 100, 300, and 500 μm), and at each of these chloroquine concentrations, the effects of six concentrations of unlabeled verapamil (0, 15, 30, 100, 200, and 400 μm) were tested (Fig. 8*A*). The data were analyzed as outlined for the quinine and chloroquine data sets, and the results are presented in Fig. 8*B*. A partial mixed-type inhibition model was found to describe the data with high statistical confidence (Δ*AIC_c_* of 0 and Akaike weight of 0.889), and an F-test confirmed that this model was preferred over full mixed-type inhibition (Δ*AIC_c_* of 4.16 and Akaike weight of 0.111; *F* = 5.6, *p* < 10^−5^). Increasing the concentration of unlabeled verapamil decreased the apparent *V*_max_ of chloroquine transport (Fig. 9*A*) and increased the apparent *K_m_* of PfCRT^Dd2^ for chloroquine (Fig. 9*B*), with the verapamil α*K*_S_ for the chloroquine-PfCRT complex (110 ± 10 μm) ∼3-fold greater than the verapamil *K*_S_ for the empty transporter (35 ± 4 μm). Finally, we evaluated the partial mixed-type inhibition model by superimposing the experimentally derived data over a three-dimensional plot of the modeled system (Fig. 9*C*). A plot of the residual values as a function of the chloroquine or verapamil concentration (Fig. 9*D*) produced, in both cases, a relatively random distribution of points across the *x* axis. These results suggest that verapamil acts as a partial mixed-type inhibitor of chloroquine transport via PfCRT^Dd2^.

**FIGURE 10.**
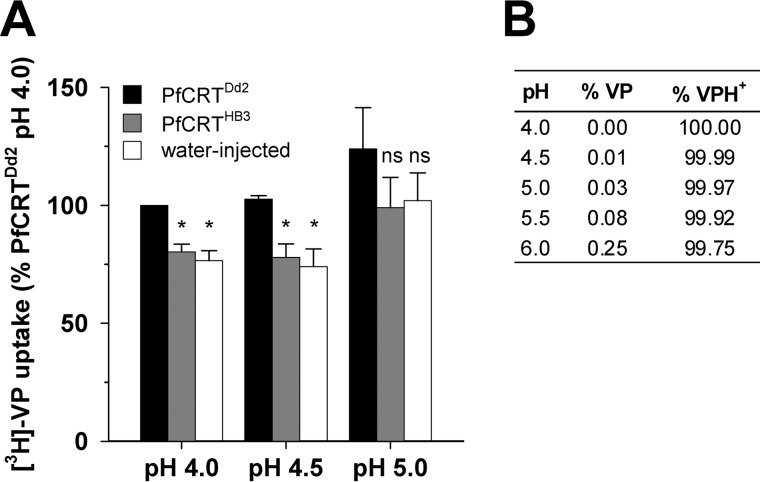
**The transport of verapamil into *Xenopus* oocytes expressing PfCRT.** *A*, the uptake of verapamil was measured in water-injected control oocytes and oocytes expressing PfCRT^HB3^ or PfCRT^Dd2^ over an extracellular pH range of 4.0–5.0. The total concentration of verapamil (radiolabeled plus unlabeled) was 5 μm. Uptake is shown as the mean ± S.E. (*error bars*) of three biological repeats, within which measurements were made from 10 oocytes/treatment. *Asterisks* indicate significant differences in the accumulation of verapamil between the control (water-injected or PfCRT^HB3^-expressing) oocytes and oocytes expressing PfCRT^Dd2^ within each pH condition (*, *p* < 0.05). *B*, the percentages of verapamil in the neutral (*VP*) and monoprotonated (*VP*^+^) forms in solutions of different pH. The percentages were calculated using the Henderson-Hasselbalch equation with a p*K_a_* value of 8.92 (52).

The ability of PfCRT^Dd2^ to translocate verapamil was investigated by measuring the uptake of radiolabeled verapamil in water-injected oocytes and in oocytes expressing PfCRT^Dd2^ or PfCRT^HB3^. As shown in Fig. 10, there was a modest but statistically significant increase in the accumulation of verapamil in oocytes expressing PfCRT^Dd2^ relative to water-injected oocytes or oocytes expressing PfCRT^HB3^. The low signal obtained for the transport of verapamil via PfCRT^Dd2^, which is most likely due to the relatively high lipophilicity of this drug (at pH 5.2 verapamil has a distribution coefficient (log *D*) of 0.52, whereas chloroquine has a log *D* of −3.44; (39)), precluded a kinetic analysis of verapamil transport.

## DISCUSSION

Here we provide direct evidence for the transport of quinine, quinidine, and the resistance reverser verapamil via PfCRT^Dd2^. By contrast, these drugs do not appear to be substrates of the wild-type form of the protein (PfCRT^HB3^). These observations are consistent with the findings of Lehane *et al.* (19, 21) as well as those of Wellems and colleagues (22, 23). The former studies showed that chloroquine, quinine, quinidine, and verapamil are each able to induce a proton leak from the digestive vacuole of parasites carrying PfCRT^Dd2^ but not from the digestive vacuole of an isogenic line expressing PfCRT^HB3^. In the work performed by Wellems and co-workers, the accumulation of chloroquine, quinine and quinidine was reduced in *D. discoideum* cells expressing PfCRT^Dd2^ at the endosomal membrane but was unchanged in the cell line expressing PfCRT^HB3^. Moreover, intact endosomes isolated from the PfCRT^Dd2^-expressing cells were also shown to accumulate less chloroquine and quinine compared with endosomes extracted from untransformed cells or cells expressing PfCRT^HB3^. Hence, there is now an appreciable body of evidence, obtained from three different experimental systems, which indicates that PfCRT^Dd2^ is not just a transporter of chloroquine but may be viewed as a “multidrug resistance carrier.”

**FIGURE 11.**
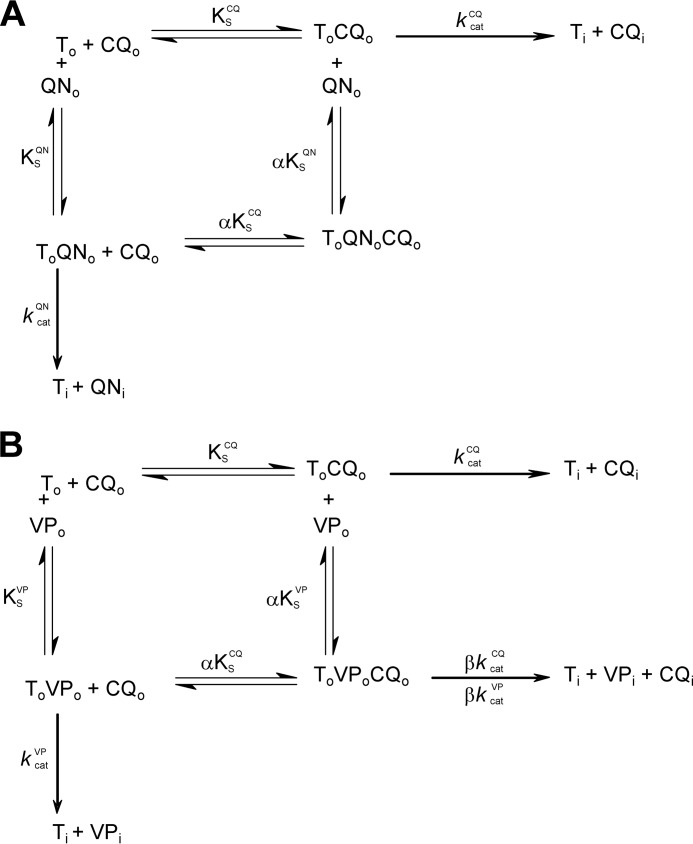
**Proposed reaction schemes describing the interaction of PfCRT^Dd2^ with chloroquine, quinine and verapamil.** *A*, chloroquine (*CQ*) and quinine (*QN*) act as full mixed-type inhibitors of PfCRT^Dd2^-mediated transport. PfCRT^Dd2^ can bind chloroquine and quinine at distinct sites. The ternary complex (*T_o_QN_o_CQ_o_*) is inactive. *T_o_* represents PfCRT^Dd2^ in the outward facing conformation (*i.e.* the extracellular face of the protein when in the oocyte plasma membrane and its luminal face when in the membrane of the digestive vacuole), and *T_i_* represents PfCRT^Dd2^ in the inward facing conformation (*i.e.* the cytosolic face of the protein when in the membrane of either the oocyte or the digestive vacuole). Likewise, *CQ_o_* and *QN_o_* are the extracellular/lumenal drug concentrations, and *CQ_i_* and *QN_i_* are the cytosolic drug concentrations. *K*_S_^CQ^ and *K*_S_^QN^ are the respective quinine and chloroquine dissociation constants for PfCRT^Dd2^; α is the factor by which *K*_S_^CQ^ and *K*_S_^QN^ change when the other drug is already bound to the transporter; and *k*_cat_^CQ^ and *k*_cat_^QN^ describe the rate constants of chloroquine and quinine transport. *B*, chloroquine and verapamil (*VP*) act as partial mixed-type inhibitors of PfCRT^Dd2^-mediated transport. The ternary complex (*T_o_VP_o_CQ_o_*) retains a low level of activity. *VP_o_* and *VP_i_* are the extracellular/lumenal and cytosolic concentrations of verapamil, respectively. *K*_S_^VP^ is the verapamil dissociation constant for PfCRT^Dd2^; α is the factor by which *K*_S_^CQ^ and *K*_S_^VP^ change when the other drug is already bound to the transporter; *k*_cat_^VP^ describes the rate constant of verapamil transport; and β is the factor by which verapamil and chloroquine affect the rate constant of the other drug's transport.

Our detailed kinetic analyses of PfCRT-mediated drug transport in the presence of a second (inhibiting) drug indicated that these drugs interact with PfCRT^Dd2^ in a complex manner. In three separate sets of experiments (Figs. 2, 4, and 8), the interactions of chloroquine, quinine, and verapamil with PfCRT^Dd2^ were best explained by models of mixed-type inhibition. This form of inhibition, which combines features of both competitive and uncompetitive inhibition, is characterized by systematic variations in the *K_m_* and *V*_max_ for the transport of one substrate in the presence of a second substrate. In all three experiments, occupation of PfCRT^Dd2^ by one drug reduced the protein's affinity for the second drug by a factor of 2–3, a finding that is consistent with the drugs acting to compete with one another's transport. However, the binding of one drug did not fully exclude the binding of the second, and the binding of both drugs to PfCRT^Dd2^ led to the formation of a ternary complex, the properties of which differed depending on the substrates that were bound. In the case of chloroquine and quinine, the value derived for the parameter β (a measure of the contribution of the chloroquine-quinine-PfCRT^Dd2^ complex to the total rate of transport), was not different from zero (Table 2). This finding indicates that the binding of both chloroquine and quinine to PfCRT^Dd2^ results in an inactive transporter and that the kinetic data are therefore better described by full mixed-type inhibition rather than partial mixed-type inhibition (Fig. 2*B*). By comparison, analyses of the kinetics of verapamil inhibition suggested that the verapamil-chloroquine-PfCRT^Dd2^ complex remains active, albeit with a chloroquine transport rate that is 30-fold lower than that measured in the absence of verapamil (β = 0.03 ± 0.01; Table 2). Hence, partial mixed-type inhibition was found to be more plausible than full mixed-type inhibition for modeling the inhibition of chloroquine transport by verapamil (Fig. 8*B*). The very low level of activity detected for the verapamil-chloroquine-PfCRT^Dd2^ complex suggests that the transporter is occasionally able to translocate chloroquine and verapamil in symport, which is a phenomenon that is unlikely to occur when chloroquine and quinine are the bound substrates. The reaction schemes describing the interactions of PfCRT^Dd2^ with (i) chloroquine and quinine and (ii) chloroquine and verapamil are shown in Fig. 11, *A* and *B*, respectively.

Very few studies have attempted to elucidate the mechanism by which resistance-conferring forms of PfCRT interact with different substrates or inhibitors. In a detailed analysis of structure-activity relationships among a series of chemosensitizing agents, Alibert *et al.* (26) predicted the features of a putative drug-binding site that would account for the patterns of resistance-reversing activity that they observed. The authors suggested that the amino groups present in many quinoline antimalarials and reversing agents (including chloroquine and verapamil) would form hydrogen bonds with a hydroxyl group of a serine and a salt bridge with the carboxyl group of an aspartic acid. They proposed that the N75E and K76T mutations in PfCRT^Dd2^, (which introduce a glutamate and a threonine residue, respectively) could fulfill these roles, forming a site of interaction where compounds compete for binding and translocation (26, 27). Studies of laboratory-derived drug-resistant parasites as well as of transgenic lines that differ in the haplotype of PfCRT they carry have provided good support for the idea that position 76 plays a crucial role in determining the susceptibility of the parasite to chloroquine and quinine (11, 28, 29). Indeed, we recently showed that the minimum changes required to enable PfCRT to transport chloroquine are K76T with either N75E or N326D (25). When considered together, these observations suggest that the threonine at position 76 is the key element of the binding site of resistance-conferring forms of PfCRT. However, the results of our kinetic analyses indicate that interactions at a single site are not sufficient to explain the complex interplay observed between PfCRT^Dd2^ and chloroquine, quinine, and verapamil. In regard to chloroquine and quinine, the mixed-type inhibition observed for these two substrates could not be attributed to a special case of product inhibition, and the results of the competition plot confirmed that these two drugs do not compete for binding at a single site. The lack of a robust signal for the uptake of verapamil via PfCRT^Dd2^ precluded a similar examination of the interplay between chloroquine and verapamil. Nevertheless, the finding that the partial mixed-type model best describes the inhibition of PfCRT^Dd2^ by verapamil suggests that chloroquine and verapamil are also likely to be binding to distinct sites. Taken together, our studies suggest that a more nuanced model is required to describe the interaction of PfCRT^Dd2^ with its substrates.

We recently showed that several of the eight mutations found in PfCRT^Dd2^ demonstrate epistasis and that the protein acquires the ability to transport chloroquine via a rigid process (whereby mutations must be added in a specific order to avoid significant reductions in chloroquine transport activity) (25). These findings indicate that the repurposing of PfCRT for the binding and translocation of chloroquine entails dramatic and complex rearrangements to its substrate-binding site (25). The work presented here suggests that these changes have also modified the transporter's ability to interact with related drugs (quinine and quinidine) as well as a relatively different pharmacon (verapamil). Bearing this in mind and given that our data indicate that PfCRT possesses distinct binding sites for chloroquine and quinine (and for chloroquine and verapamil), it is possible that the mutations required for chloroquine resistance have enlarged the protein's substrate-binding site by creating new substrate interaction points and/or multiple binding pockets. A large drug-binding cavity consisting of different substrate interaction domains has been observed in several other membrane transport proteins, including the multidrug resistance P-glycoprotein and the organic cation transporters (40–43).

There are several ways in which the binding of a drug at one pocket of PfCRT^Dd2^ could influence the properties of other substrate-binding sites. First, the binding of one substrate could result in conformational changes that alter the protein's ability to bind a substrate at a second site. This allosteric mechanism is consistent with the induced fit model of protein function, which has been remarkably successful in explaining the functions of a range of proteins, including transporters and channels (44). Our observations could also be reconciled with a slightly different scenario in which chloroquine and other compounds interact with several points of attachment within a large substrate-binding cavity. If one or more of these points of attachment are used by both of the substrates, then binding could be partial or reduced in affinity or could result in a shift in location of one substrate by the other, and it may even fail to trigger translocation. In this regard, it is worth noting that partially overlapping binding sites are often found in cases of mixed-type inhibition (43, 45, 46).

A third mechanistic explanation for the effect of a bound drug on a second binding site comes from the observation that PfCRT^Dd2^ appears to transport drugs in their mono- and/or diprotonated forms (24) (Figs. 1*A* and 10*B*). The attachment of a protonated drug would add one or more positive charges to the protein's substrate-binding cavity, resulting in a reduction in the electrostatic attractions between this domain and the second protonated drug. In this regard, it is worth noting that of the eight amino acids that differ between PfCRT^HB3^ and PfCRT^Dd2^, four result in the gain of a negative charge or the loss of a positive charge (N75E, K76T, Q271E, and R371I). Hence, it is highly likely that electrostatic interactions play an important role in the transport activity of PfCRT^Dd2^. This electrostatically driven model of inhibition is similar to the one proposed by Warhurst (39), who suggested that verapamil (in its protonated form) reduces the transport of chloroquine via PfCRT^Dd2^ by restoring the positive charge that was removed by the K76T mutation.

Our findings have a number of implications for the design and development of new antimalarial drugs and strategies. We have recently shown that the transport of chloroquine via a wide range of resistance-associated isoforms of PfCRT is saturable, and, in parasites exposed to a standard clinical dose of chloroquine, the transporter would already be operating at or near maximum capacity (25). These observations, together with a recent clinical trial that revealed that “double dose” chloroquine is as effective as the current gold standard antimalarial (artemether-lumefantrine) in treating *P. falciparum* infections in the Republic of Guinea-Bissau (47), have raised the very real possibility that chloroquine-resistant malaria from regions around the world could likewise be treated by a revised dosage of the drug. The work presented here has extended these observations by demonstrating that the transport of quinine and quinidine via PfCRT^Dd2^ is also saturable. This finding adds further support to the view that PfCRT^Dd2^ behaves as a multidrug resistance carrier rather than as a channel. Moreover, it may explain, at least in part, why revisions of the quinine dose regimen (which have entailed increases in the quantity of quinine per dose, the frequency of administration, and/or the duration of the course) have largely resulted in significantly improved cure rates in regions where drug-resistant malaria is prevalent (reviewed in Ref. 48). Hence, it is becoming increasingly apparent that the saturability of drug transport via PfCRT is likely to represent a potential “Achilles' heel” of the quinoline resistance mechanism and that antimalarial drugs (existing and future) for which clinical efficacy is compromised by mutations in PfCRT may be restored to efficacy by using reoptimized dosages.

The results of our kinetic analyses also indicate that different drugs are likely to interact with distinct sites within a polyspecific substrate recognition cavity of PfCRT^Dd2^, resulting in different levels of affinity and transport efficiency. This suggests that there may be scope for the interaction of an antimalarial drug with PfCRT^Dd2^ to be minimized or even eliminated with modest modifications to its structure, thus allowing the drug to escape the resistance mechanism altogether. In other cases, it may be desirable to increase and optimize the interactions between PfCRT^Dd2^ and a drug. For instance, a clinically effective reverser of chloroquine resistance is likely to be a drug that occupies multiple sites within the PfCRT^Dd2^ substrate-binding cavity, because increasing the number of attachment points between the inhibitor and the transporter should increase the affinity of binding. Furthermore, once an inhibitor of this type is bound to the protein at one site, the probability of another part of the molecule binding to a second site is substantially increased relative to two separate compounds binding to the same sites (49). In this regard, it is interesting to note that a series of quinine dimers have recently been reported as the most potent inhibitors of PfCRT^Dd2^ identified to date (50). These compounds inhibited the PfCRT^Dd2^-mediated transport of chloroquine into *Xenopus* oocytes with half-maximum inhibitory concentrations between 1 and 6 μm. These values are substantially lower than that measured for the quinine monomer (48 ± 3 μm (24)). Moreover, the quinine dimers were not substrates of PfCRT^Dd2^, and their strong affinity for the transporter translated into potent resistance-reversing activity. For example, low nanomolar concentrations of a quinine dimer restored the activity of chloroquine against chloroquine-resistant parasites, whereas micromolar concentrations were required for verapamil to exert an effect. The potent activity of the quinine dimers against PfCRT^Dd2^ is probably due to their occupation of more than one site within the transporter's substrate-binding cavity.

The work described here provides direct evidence of the ability of a chloroquine resistance-conferring form of PfCRT to transport quinine, quinidine, and verapamil. In a series of detailed kinetic analyses, we showed that chloroquine and quinine and also chloroquine and verapamil are mixed-type inhibitors of one another. The patterns of inhibition that we observed indicate that PfCRT^Dd2^ possesses more than one drug-binding site and that these binding sites, although distinct, are interdependent and hence may be located within a large polyspecific substrate recognition cavity. Our findings suggest that the mutations that confer resistance to chloroquine allow the protein to interact with and transport a range of structurally diverse compounds. However, the additional substrate attachment points generated by these changes could be exploited for the design of potent resistance-reversing agents.

## Footnotes

5 The abbreviation used is:

PfCRT: *P. falciparum* chloroquine resistance transporter.
